# Mismatch Negativity/P3a Complex in Young People with Psychiatric Disorders: A Cluster Analysis

**DOI:** 10.1371/journal.pone.0051871

**Published:** 2012-12-14

**Authors:** Manreena Kaur, Jim Lagopoulos, Philip B. Ward, Tamara L. Watson, Sharon L. Naismith, Ian B. Hickie, Daniel F. Hermens

**Affiliations:** 1 Clinical Research Unit, Brain and Mind Research Institute, the University of Sydney, Sydney, Australia; 2 School of Psychiatry, University of New South Wales, Kensington, New South Wales, Australia; 3 Schizophrenia Research Unit, South West Sydney Local Health Network, Liverpool Hospital, Liverpool, New South Wales, Australia; 4 School of Social Science and Psychology, University of Western Sydney, Kingswood, New South Wales, Australia; Baylor College of Medicine, United States of America

## Abstract

**Background:**

We have recently shown that the event-related potential biomarkers, mismatch negativity (MMN) and P3a, are similarly impaired in young patients with schizophrenia- and affective-spectrum psychoses as well as those with bipolar disorder. A data driven approach may help to further elucidate novel patterns of MMN/P3a amplitudes that characterise distinct subgroups in patients with emerging psychiatric disorders.

**Methods:**

Eighty seven outpatients (16 to 30 years) were assessed: 19 diagnosed with a depressive disorder; 26 with a bipolar disorder; and 42 with a psychotic disorder. The MMN/P3a complex was elicited using a two-tone passive auditory oddball paradigm with duration deviant tones. Hierarchical cluster analysis utilising frontal, central and temporal neurophysiological variables was conducted.

**Results:**

Three clusters were determined: the ‘globally impaired’ cluster (n = 53) displayed reduced frontal and temporal MMN as well as reduced central P3a amplitudes; the ‘largest frontal MMN’ cluster (n = 17) were distinguished by increased frontal MMN amplitudes and the ‘largest temporal MMN’ cluster (n = 17) was characterised by increases in temporal MMN only. Notably, 55% of those in the globally impaired cluster were diagnosed with schizophrenia-spectrum disorder, whereas the three patient subgroups were equally represented in the remaining two clusters. The three cluster-groups did not differ in their current symptomatology; however, the globally impaired cluster was the most neuropsychologically impaired, compared with controls.

**Conclusions:**

These findings suggest that in emerging psychiatric disorders there are distinct MMN/P3a profiles of patient subgroups independent of current symptomatology. Schizophrenia-spectrum patients tended to show the most global impairments in this neurophysiological complex. Two other subgroups of patients were found to have neurophysiological profiles suggestive of quite different neurobiological (and hence, treatment) implications.

## Introduction

A major topic of debate in psychiatric research is whether categorical diagnoses (e.g. depressive disorder, bipolar disorder and schizophrenia) represent distinct disorders (in terms of their underlying neurobiology) or, if they are better represented as a continuum of psychiatric illness [1]–[3]. Emerging neurobiological evidence suggests that these separately categorised disorders share more in common than previously purported [4]–[11] and that endophenotypes are ideally placed to investigate the underlying aetiology [8], [12], [13]. Moreover, this corpus of work suggests a better understanding of these disorders may be achieved using a ‘bottom up’ approach utilising endophenotypes rather than traditional ‘top down’ methods (based on symptomatology or diagnoses). Data driven methods have the capacity to delineate novel findings in cohorts of patients with emerging psychiatric symptoms, given the potentially variable longitudinal trajectories in such patients.

Over the past decade, impaired mismatch negativity (MMN) and P3a, have been established as endophenotypes (or biomarkers) of schizophrenia [14]–[17]. Both of these endophenotypes (but MMN in particular) have been extensively studied in schizophrenia and have shown to be highly reliable over time, resilient to practice effects, relatively independent of fluctuations in clinical features, heritable, and when compared to controls, exhibit large effect size deficits [8], [13], [14], [18]. This research suggests that as endophenotypes, MMN and P3a are robust and have utility in probing the underlying neurobiology, and therefore clinical and functional outcomes in complex diseases such as schizophrenia and bipolar disorder. In deviance detection paradigms, these event-related potentials (ERPs) co-occur, are sequential [19] and have been described as the ‘MMN/P3a complex’ [4], [5], [20]–[22]. MMN is thought to emerge from frontal and temporal brain generators and it indexes the brain’s ability to extract relevant information from an irrelevant background [23]–[26]. P3a is generated fronto-centrally and reflects a subsequent reorienting process [27]. There is an extensive literature showing associations between MMN and cognition [4], [21], [28]–[31] as well as with psychosocial functioning [5], [21], [22], [28], [32]–[35], whereby smaller MMN amplitudes correspond with poorer levels of cognitive/psychosocial functioning. Similar associations have been found between P3a amplitudes and cognitive or psychosocial functioning [21], [22] suggesting that the MMN/P3a complex may be a marker of the fundamental sensory processes that underlie higher-order functions. Recently, studies have shown that these ERPs may not be as specific to schizophrenia as initially considered. MMN is thought to reflect NMDA receptor mediated neurotransmission and therefore impaired MMN is likely to reflect glutamatergic dysfunction [36], [37], whereas variations in the amplitude of P3a are thought to be primarily modulated by dopaminergic changes [38], [39]. Both of these neurotransmitters have been shown to be dysregulated across several psychiatric disorders, making them ideal for exploration in this cohort.

Our group has reported on the MMN/P3a complex in first-episode psychosis, early psychosis subgroups (i.e. schizophrenia-spectrum and affective-spectrum) and early bipolar-spectrum disorders (with and without psychotic symptoms) [4], [5], [21]. We have demonstrated that the MMN/P3a complex in early affective-spectrum disorders is similarly impaired as in early schizophrenia-spectrum disorders. Other studies have corroborated our findings and have similarly reported impairments in the amplitudes of these biomarkers in major depression and bipolar disorder [7], [19], [40]–[43]. There is now a confluence of evidence across a range of psychiatric disorders reporting similar deficits in the MMN/P3a complex suggesting that commonalities may exist in neuropathological processes of early psychiatric illnesses.

In light of the aforementioned findings, a data driven approach for investigating differences in MMN/P3a in a cohort of young people with an admixture of psychiatric disorders may prove insightful. Accordingly, we utilised cluster analysis, a hypothesis generating and exploratory analysis, to determine unique neurophysiological profiles in young psychiatric patients and examined whether these profiles could explain demographic, clinical and/or cognitive differences. We hypothesised that distinct MMN/P3a profiles would exist despite the clinical and cognitive features of these patients.

## Methods

The study and consent procedure was approved by the University of Sydney Human Research Ethics Committee. All participants were determined by their referring psychiatrist to have the mental and intellectual capacity to give written informed consent prior to participation in the study. All participants were aged 16 years or older and were able to give their own written informed consent (i.e. parental/guardian consent is not required for those aged 16 and above according to the University of Sydney Human Research Ethics Committee guidelines and Australian law) prior to participation in the study.

### Participants

Eighty-seven outpatients (16 to 30 years) with an admixture of psychiatric disorders were recruited from specialised referral services for the assessment and early intervention of mental health problems [44], [45]. Initial diagnoses were determined by a referring psychiatrist, according to DSM-IV criteria [46]. Subsequently, a research psychologist conducted a structured interview and case-review (under the supervision of a psychiatrist or clinical psychologist) utilising the psychosis and mood disorders section of the Structured Clinical Interview for DSM-IV [47] to confirm the diagnoses as follows: depressive disorders [major depressive disorder (n = 12); major depressive disorder with psychotic features (n = 7)]; bipolar disorders [bipolar I (n = 6); bipolar II (n = 6); bipolar I with psychotic features (n = 10); bipolar II with psychotic features (n = 4)]; psychotic disorders [schizophrenia (n = 24); schizophreniform (n = 10); schizoaffective (n = 8)]. Patients were tested under ‘treatment-as-usual’ conditions, and medication regimens were not interrupted. Sixty-three patients were on an antipsychotic, 34 were on an antidepressant, 20 were on a mood stabiliser and 7 were on other psychotropic medication (i.e. benzodiazepines or stimulants).

The healthy control group (n = 27; aged 16 to 30 years; 14 females) was recruited from the community in the same region and were screened for psychopathology by a research psychologist via clinical interview. Exclusion criteria for all participants were diagnosis of a substance use disorder, medical instability, history of neurological disease, medical illness known to impact brain function, intellectual and/or developmental disability and insufficient English for assessment. In addition to this, control participants with a family history of a psychotic- or bipolar-disorder were excluded. All participants were asked to abstain from illicit drug or alcohol use for 48 hours prior to testing. To verify recent abstinence, participants also underwent an alcohol breath test and a saliva drug screen to determine presence of cannabinoids, meth/amphetamines, opiates, benzodiazepines and cocaine. None of the participants were intoxicated at the time of testing; if any of the participants failed the drug screening, their assessment was cancelled and they were assessed on another day.

### Clinical and Neuropsychological Assessment

To quantify current symptoms, a research psychologist made clinical ratings using the Hamilton Depression Rating Scale (HDRS, 17-item) [48] and the Brief Psychiatric Rating Scale (BPRS) [49]. Positive and negative symptom sub-scores of the BPRS were also calculated [50]. Participants were rated on the social and occupational functioning assessment scale (SOFAS) [51]. Premorbid intelligence (‘predicted IQ’) was estimated based on performance on the Wechsler Test of Adult Reading [52]. Processing speed was assessed using the Trail-Making Test, part A (TMT A) [53]; with set-shifting assessed by part B (TMT B). Verbal learning and memory were assessed via the Rey Auditory Verbal Learning Test (RAVLT); variables assessed were: immediate recall (sum of trial 1–5; RAVLT A1 to A5) and 20-minute delayed recall (trial 7; RAVLT A7). Patients were asked to complete a self-report assessment which included the Kessler-10 (K-10) measuring psychological distress [54] and the Alcohol Use Disorder Identification Test (AUDIT) to determine harmful levels of alcohol use [55].

### Neurophysiological Testing

After preparation for EEG recording, participants were presented (via headphones) with 2,500 binaural pure tones (1,000 Hz, 75 dBSPL, 10 ms rise/fall) at a 500 ms stimulus onset asynchrony; this comprised a pseudo-random sequence of 2,300 (92%) 50 ms standard tones and 200 (8%) 100 ms deviant tones. Tones were presented while participants watched a silent movie and subjects were asked to report back the storyline of the movie at the end of the task. A 64-channel Quik-Cap (Neuroscan) acquired EEG data from sites according to the standard 10–20 International system (including mastoids). Data was referenced to a nose electrode. Vertical and horizontal electro-oculogram was monitored for eye-blink artefacts and contaminated data was corrected using established algorithms [56]. The mean amplitude, peak amplitude and peak latency was determined for MMN and P3a, according to established epoch windows of 135–205 ms and 250–300 ms, respectively [4], [5], [18], [21], [22], [29], [32]–[34], [42]. Scalp and EOG potentials were digitised continuously at 500 Hz and signal processing was performed offline using Neuroscan Scan 4.3.1 (Compumedics) software. Data were filtered using a bandpass filter (0.15–20 Hz) and epochs of EEG that were contaminated by movement artefacts (±100 µV) were rejected.

### Statistical Analyses

Statistical analyses were performed using SPSS for Windows 20.0. To facilitate interpretation of ERP data in the cluster analysis (see below), values for MMN amplitude at Fz were multiplied by −1.0 so that the changes in amplitudes were the same across the three variables (i.e. MMN at Fz, MMN at M1 and P3a at Cz). Following this, to achieve consistency within the cluster analysis all three neurophysiological variables were converted to ‘standardised’ values. A hierarchical cluster analyses utilising Wards method of minimum variance with a squared Euclidean distance measure was conducted to identify variation in patterns among neurophysiological variables. Typically, Fz and Cz as well as left and right temporal sites (i.e. M1 and M2) have been examined in the same studies. However, these pairs of variables are often highly correlated, therefore, only one (from each ‘pair’) was chosen for each component in order to circumvent any redundancy in the cluster analysis. Firstly, a number of studies have reported that patients have greater impairments in left, rather than right, temporal MMN [5], [57], [58]. Secondly, imaging studies of psychosis patients indicate greater reductions in left temporal volumes [59]–[61]. For these reasons MMN at M1 (and not M2) was selected. Finally, P3a at Cz was selected (rather than Fz) as this component tends to be maximal at the vertex with increased sensitivity in distinguishing patient groups [4], [5], [21].

Cluster analysis was based on previous similar studies [6], [62]–[64] and statistical recommendations [65]. Cluster analysis is a classification technique for forming homogeneous groups within complex data sets and the aim of the present study was to determine whether a sample of young (16–30 yrs old), psychiatric outpatients would form clusters on the basis of their neurophysiological profiles. A healthy control group was intentionally omitted from the analysis as they are clearly different and to include them in a cluster analysis would have led to an unnecessary re-distribution of the results, in accordance with established literature [6], [63], [66], [67].

One-way between-subject analysis of variance (ANOVA) was used to assess differences in the (uncorrected) neurophysiological measures as well as in the demographic, clinical and neuropsychological variables among cluster groups. The chi-square test was used to compare the ratio of females to males across groups. Significance levels were set at p<0.05. Levene’s test was used to test for homogeneity of variance. Welch’s statistic was calculated, with corrected df and p-values reported, where this assumption was violated. For pair-wise cluster group comparisons post-hoc Scheffe’s test were employed. A confirmatory discriminant function analysis (DFA) was performed to determine which neurophysiological variables best distinguished the cluster groups. As a secondary analysis, separate ANOVAs were used to assess differences between the cluster groups and controls with post-hoc Dunnett’s tests to determine each cluster group-to-control comparison.

## Results

### Cluster Characteristics

**Figure 1:**
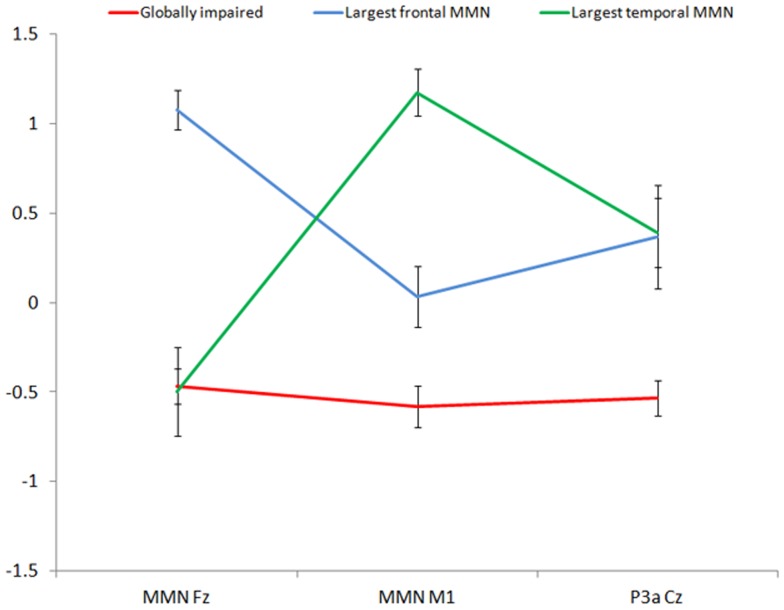
Profile of standardised mean amplitude values (with standard error bars) for mismatch negativity (MMN) and P3a for the ‘globally impaired’ (red), ‘largest frontal MMN’ (blue) and ‘largest temporal MMN’ (green) cluster groups. Event-related potential mean amplitudes (µV) were standardised and corrected (for consistency in polarity between variables) so that positive values reflect increased amplitudes.

Agglomeration coefficients generated by cluster analysis revealed a demarcation point between three- and four- cluster solutions, suggesting that a three-cluster solution best distinguished the cases; this was confirmed by inspection of the dendrogram. The resultant clustering revealed three relatively well-sized groups which were labelled according to their most distinguishing characteristic; the first is the ‘globally impaired’ cluster (n = 53), the second is the ‘largest frontal MMN’ (+FMMN) cluster (n = 17) and the third is the ‘largest temporal MMN’ (+TMMN) cluster (n = 17). Table 1 shows the cluster group mean amplitude (µV) and standard deviations for each of the three (unstandardised) neurophysiological variables. As revealed by ANOVA (see Table 1), for each neurophysiological measure there was a significant (p<.001) main effect of cluster group. Post-hoc comparisons further revealed significant differences between cluster pairs. The globally impaired and +FMMN clusters were differentiated by all three neurophysiological variables; that is, frontal and left temporal MMN, and P3a amplitudes (p<.05), with the globally impaired cluster showing reduced amplitudes across each site. The globally impaired cluster was found to have significantly reduced (p<.01) left temporal MMN and P3a amplitudes compared with the +TMMN cluster. However, these clusters showed similar impairments in frontal MMN amplitudes. The +FMMN and +TMMN clusters were found to significantly differ in frontal and temporal MMN (p<.001) but not P3a amplitudes. As suggested by the overall pattern described above, these clusters showed opposing amplitudes in frontal versus temporal MMN (see Figure 1).

**Table 1:** Mean amplitudes in µV (± standard deviation) for unstandardised neurophysiological variables across the cluster and control groups (globally impaired: GI; largest frontal MMN: +FMMN; largest temporal MMN: +TMMN; controls: Cntl) with corresponding significance test values.

	GI (N = 53)	+FMMN (N = 17)	+TMMN (N = 17)	Significance Test [p]	Pair-wise comparison (p)			Cntl (N = 27)	Pair-wise comparison (p)		
					GI vs +FMMN	GI vs +TMMN	+FMMN vs +TMMN		G1 vs Cntl	+FMMN vs Cntl	+TMMN vs Cntl
MMN Fz	−3.6±1.6	−7.1±1.0	−3.6±2.2	F (2, 86) = 30.1 [.000]	†††		†††	−5.9±2.1	†††		†††
MMN M1	1.4±1.3	2.3±1.1	4.0±0.8	F (2, 86) = 34.3 [.000]	†	†††	†††	2.8±1.3	†††		††
P3a Cz	2.8±1.9	5.2±3.2	5.3±2.1	F (2, 28.1) = 12.2 [.000]	††	††		5.7±2.7	†††		

††† (p<.001), †† (p<.01) and † (p<.05) denotes significant post-hoc pair-wise comparisons derived by the Scheffe’s test (for between cluster groups) and Dunnett’s test (for between cluster groups and controls).

The neurophysiological profiles for the three cluster groups are depicted in Figure 1. The globally impaired cluster showed reductions in frontal MMN, temporal MMN and P3a amplitudes. The +FMMN cluster was distinguished by having the largest frontal MMN amplitudes with intermediate temporal MMN and P3a. Finally, the +TMMN cluster was characterised by having the most contrasting frontal as compared to temporal MMN amplitudes. That is, the +TMMN cluster showed reductions in frontal MMN but with corresponding increases in temporal MMN amplitudes (relative to the remaining clusters). For P3a, the +FMMN and +TMMN clusters showed very similar amplitudes.

As shown in Table 2, there were no significant main effects of cluster group across demographic and clinical variables. Correspondingly, the cluster-pair comparisons confirmed that there were no significant differences in terms of distribution of gender, age, age of psychiatric onset (that any psychiatric symptoms were detected), predicted IQ and functional and clinical variables.

**Table 2:** Mean values (± standard deviation) for demographic and clinical variables across the three cluster groups with corresponding significance test values.

	Globally impaired (N = 53)	Largest frontal MMN (N = 17)	Largest temporal MMN (N = 17)	Significance Test [p]
Sex (f/m)	19/34	8/9	8/9	?2 (2) = 1.1 [.582]
Age, years	22.4±4.2	23.1±3.9	21.5±2.9	F (2, 35.8) = 0.8 [.358]
Age of onset	18.1±5.5	17.6±5.4	15.9±3.8	F (2, 76) = 1.1 [.355]
Predicted IQ	100.7±8.7	105.9±7.6	100.9±12.2	F (2, 83) = 2.0 [.136]
SOFAS	60.1±12.8	58.2±8.7	59.3±10.8	F (2, 67) = 0.1 [.869]
HDRS total	9.2±6.8	11.0±9.6	11.4±7.9	F (2, 84) = 0.7 [.500]
BPRS total	38.3±12.0	38.3±12.2	45.0±15.8	F (2, 83) = 1.8 [.169]
BPRS pos	11.3±4.8	11.3±4.3	14.5±5.5	F (2, 82) = 2.8 [.069]
BPRS neg	7.9±3.4	7.3±3.1	8.9±4.0	F (2, 82) = 0.9 [.421]
K 10	23.8±9.8	28.3±10.4	24.7±9.8	F (2, 77) = 1.2 [.314]
AUDIT total	8.3±9.5	10.2±7.5	3.9±7.2	F (2, 86) = 2.4 [.096]

The Scheffe’s test was employed for post-hoc pair-wise comparisons between cluster groups, however, no significant differences were observed.

With regards to neuropsychological variables (see Table 3), there were no significant main effects of cluster group or significant cluster-pair comparisons for TMT A or TMT B. However, for RAVLT SUM and RAVLT A7, there were significant (p<.01) main effects of cluster group, with the only one significant pair-wise comparison showing that the globally impaired cluster performed worse that the +FMMN cluster in RAVLT A7.

**Table 3:** Mean values (± standard deviation) for neuropsychological variables across the cluster and control groups (globally impaired: GI; largest frontal MMN: +FMMN; largest temporal MMN: +TMMN; controls: Cntl) with corresponding significance test values.

	G1 (N = 53)	+FMMN (N = 17)	+TMMN (N = 17)	Significance Test [p]	Pair-wise comparison (p)			Cntl (N = 27)			
					G1vs +FMMN	GI vs+TMMN	+FMMN vs +TMMN		G1 vs Cntl	+FMMN vs Cntl	+TMMN vs Cntl
TMT A	31.0±9.4	24.7±10.1	32.4±15.6	F (2, 82) = 2.2 [.121]				23.3±4.9	††		†
TMT B	74.3±31.7	61.5±12.4	68.4±26.7	F (2, 34.4) = 1.3 [.077]				52.3±15.5	††		
RAVLT sum	50.2±10.8	57.7±4.5	48.9±13.5	F (2, 32.1) = 3.4 [.001]				59.3±5.1	†††		††
RAVLT A7	9.8±3.7	12.6±2.1	10.9±3.6	F (2, 31.7) = 4.0[.003]	†			13.4±1.3	†††		†

††† (p<.001), †† (p<.01) and † (p<.05) denotes significant post-hoc pair-wise comparisons derived by the Scheffe’s test (for between cluster groups) and Dunnett’s test (for between cluster groups and controls).

### Relationship between Cluster Membership and Primary Diagnosis

The distribution of primary diagnoses among the cluster groups are presented in Table 4. In terms of primary diagnoses, just over half of the patients within the globally impaired cluster had a psychotic disorder (55%) while patients with bipolar versus depressive disorders were equally represented (25% vs. 21%, respectively). Notably, all three psychiatric groups were more equally distributed among the +FMMN and the +TMMN clusters (see Table 4). The medication status of patients in each cluster is summarised in Table 5. The prevalence of ‘any’ anti-psychotic, anti-depressant, mood stabiliser or ‘other’ medication was relatively balanced across the three cluster groups. Chi-square tests revealed no significant differences in the presence (or not) of each diagnostic or medication category among the three cluster groups (all p>.05).

**Table 4:** Cross-tabulation of cluster by primary diagnosis.

Primary Diagnosis		Globally impaired	Largest frontal MMN	Largest temporal MMN
Depression	Count	11	5	3
	%	21%	29%	18%
Bipolar	Count	13	6	7
	%	25%	35%	41%
Psychosis	Count	29	6	7
	%	55%	35%	41%

**Table 5:** Cross-tabulation of cluster by medication category; Note: the ‘other psychotropic’ medications category includes benzodiazepines or stimulants.

Current Medication		Globally impaired	Largest frontal MMN	Largest temporal MMN
Anti-psychotic	Count	41	11	11
	%	77%	65%	65%
Anti-depressant	Count	18	10	6
	%	34%	59%	35%
Mood-stabiliser	Count	12	5	3
	%	23%	29%	18%
Other psychotropic	Count	3	2	2
	%	6%	12%	12%

### Discriminant Function Analysis

With the three neurophysiological variables entered as predictors, DFA confirmed the distinct profiling by generating two functions to separate the 3 cluster-groups. The first function accounted for 78.5% of the differences among the clusters [Wilk’s λ = 0.221, p<.001]. The second function explained the remaining variance (21.5%) and was also statistically significant [Wilk’s λ = 0.652, p<.001]. As revealed by the structure matrix, the first function had a high discriminant loading for MMN at M1 (r = .523) whereas the second function had high discriminant loadings for MMN at Fz (r = .873) and MMN at M1 (r = .729).

### Comparison with Healthy Controls

As a secondary analysis, we sought to determine whether any of the neurophysiological variables determined for each cluster group were significantly abnormal compared to healthy controls. Chi-square test confirmed that there was no difference in the ratio of females-to-males between controls (14F: 13M) and the cluster groups. Dunnett’s post hoc tests determined that there were no significant differences between any of the cluster groups and controls in terms of mean age (controls: 23.1±3.3 years). However, despite each cluster group having a mean predicted IQ score above 100, the control group had significantly higher IQ scores compared to the globally impaired (p<.01) and the +TMMN (p<.05) clusters.

**Figure 2:**
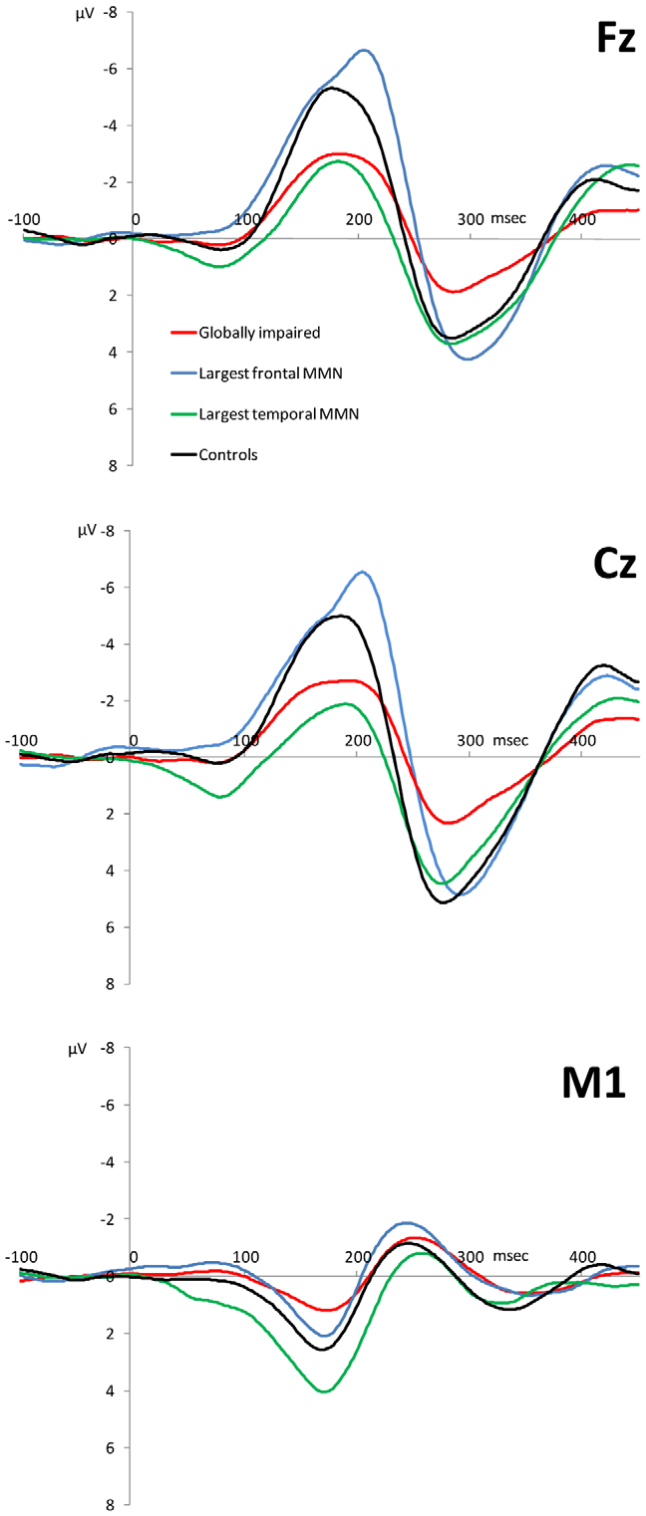
Grand average event-related potentials for the ‘globally impaired’ (red), ‘largest frontal MMN (blue)’, ‘largest temporal MMN’ (green) cluster and control (black) groups at (from top to bottom) frontal (Fz), central (Cz) and left temporal (M1) sites. *Note*: M1 waveforms are reversed in polarity due to the nose-referenced recording.

As shown in Table 1 there was a range of significant pair-wise (i.e. Dunnett’s) comparisons across the (uncorrected) neurophysiological variables. ANOVA confirmed that there were no between-group differences in the number of epochs accepted for each cluster group and the controls (p = 0.16). The globally impaired cluster showed significant (p<.001) deficits in all three neurophysiological variables as compared to controls, supporting the global impairment finding described above. In contrast, the +FMMN cluster did not differ significantly from controls. Finally, the +TMMN cluster showed significant differences from controls across MMN but not P3a variables; with reduced (at p<.001) frontal MMN but increased (at p<.01) temporal MMN amplitudes. Figure 2 illustrates the grand average MMN (frontal and temporal) and P3a waveforms for each cluster group as compared to controls.

There were differences between the cluster groups and controls across all neuropsychological variables. Only the globally impaired cluster exhibited reduced cognitive functioning across all measures. More specifically, the globally impaired cluster had significantly poorer processing speed and set-shifting (both p<.01) as well poorer verbal learning and memory (both p<.001). Notably, the +TMMN cluster performed poorer than controls in verbal learning and memory as well as processing speed (all p<.05). There were no significant differences in the neuropsychological measures between the +FMMN cluster and controls.

## Discussion

The results from our data indicate that three distinct neurophysiological profiles are evident in our patient population. The largest subgroup that was identified is characterised by a global impairment of the MMN/P3a complex, as reflected by the reductions in frontal and temporal MMN and P3a amplitudes. The +TMMN cluster bears similarities to the globally impaired cluster in terms of deficits in frontal MMN amplitudes, however was unique from the other two clusters by exhibiting the largest temporal MMN amplitudes. Finally, the +FMMN cluster was distinguished from the other clusters in that it had the largest frontal MMN amplitudes. Notably, no differences were observed between all three cluster-groups for any clinical and cognitive measures except that the globally impaired cluster displayed poorer verbal memory compared to the +FMMN cluster.

Just over half (55%) of the patients in the globally impaired cluster comprised of individuals with a primary diagnosis of a psychotic disorder. Furthermore, the global impairments identified in this cluster are consistent with the extant literature which reports that deficits in frontal and temporal MMN amplitudes are well established findings in schizophrenia, and more recently, earlier stages of psychotic disorders [4], [5], [15], [21], [23], [31], [43], [57], [58], [68]. Moreover, it is well established that these neurophysiological components reflect the integrity of the neural pathways that they represent and as such, the relative health of these pathways can be gleaned via measures such as amplitude as well as from the corresponding morphology of these signals [12], [36]. In the case of the globally impaired cluster, the observed decrement in amplitudes for both temporal (MMN) and frontal (MMN) signals suggests that for these patients there may be a global disruption in the MMN subsumed by deficits in both the sensory memory and the automatic attention-switching mechanisms underlying deviance detection [25], [26], [69]. Furthermore, this neurophysiological profile corresponded with the worst overall cognitive profile; a finding that is consistent with significant associations reported between MMN and a range of cognitive variables [4], [21], [22], [28]–[31]. This association should be treated with caution as although the globally impaired cluster was the only group to show significant reduction across all neuropsychological measures compared to controls, there was only one significant difference among the cluster groups (see pair-wise comparisons in Table 3). Potentially, such functional associations may be long-term, with other evidence showing that patients with the greatest MMN impairments at baseline had the worst functional outcomes at follow-up [59].

These findings indicate that a subgroup of patients, at the early stages of a psychotic disorder, already display significant neurobiological impairment. In other words, a global reduction in MMN/P3a with corresponding impairments in cognitive performance may be a biomarker for a more distinct and/or prolonged psychotic (or related) illness. However, it is important to keep in mind that 46% of the globally impaired cluster consists of individuals with a primary affective disorder (i.e. a depressive or a bipolar disorder with and without psychotic features). It is possible that, especially given their younger age, that some of these patients may be on a pathway to a psychotic illness (with the depressive phenotype currently dominating). Notwithstanding, impaired MMN and P3a has been documented in individuals with affective disorders [4], [5], [7], [9], [43] and a recent study by our group has reported impaired temporal MMN in an older sample of patients with late-life depression indicating a specific disruption in temporal networks [70]. Similarly, in psychotic disorders, significant impairments in temporal MMN tend to be found in chronic samples [57], [58], [68]. Critically, no studies have undertaken a data-driven approach to investigate the extent to which there may be subsets of patients with broad (in particular, temporal) impairments in MMN so it is difficult to determine whether global impairments in MMN/P3a represent severity, chronicity or both. In all likelihood, the neurophysiological profile shown by the globally impaired cluster may not be related to a particular diagnosis or syndrome but rather represent more severe deficits that are indicative of a developmental origin and related to general brain dysfunction. As such, this may be a larger risk factor for psychotic (and related) disorders, but not exclusively.

The +TMMN cluster bears similarities to the globally impaired cluster in that they both show deficits in frontal MMN. Frontal MMN impairments have been reported in both chronic schizophrenia and also early stages of psychotic illnesses, such as first-episode psychosis and ultra-high risk for psychosis [21], [71]–[73]. On the other hand, only one study has reported impairments in temporal MMN at early stages of psychotic illnesses [5]; a finding which is more common in chronic schizophrenia samples [57], [58], [68]. We have proposed that deficits in temporal MMN may develop with severity and/or chronicity [4], [70]. In light of this, the absence of impairment in temporal MMN in the +TMMN cluster may indicate that individuals are less severe or at an earlier stage of their illness compared to those in the globally impaired cluster. The +TMMN cluster is equally represented by individuals with psychotic (41%), bipolar (41%) and depressive disorders (18%). Notably, Hafner and colleagues determined that the most common and stable symptoms across affective and psychotic disorders are depressive symptoms, negative symptoms and functional decline [3]. This research suggests that at early stages of both psychotic and affective disorders, the longitudinal trajectory is often unclear. Therefore, we speculate that of the individuals diagnosed with affective disorders in the +TMMN cluster (who may not currently display positive symptoms of psychosis), some might display such symptoms in the future and show more widespread neurophysiological deficits indicating further neurobiological changes.

The most surprising finding in this study was the significantly increased frontal MMN which characterised the +FMMN cluster. Increased frontal MMN is thought to reflect a hyper-glutamatergic state such as that seen in those who are prone to alcoholism [74]. Of note, this cluster had the highest, albeit non-significant, AUDIT total scores (see Table 2) suggesting some association with risky drinking. These individuals may represent a distinctly separate phenotype, despite symptomatology or diagnostic category, and may have quite different treatment implications, particularly given the recent interest in treating psychotic illnesses with glutamatergic agents [75], [76]. NMDA receptor hypo-function and dopaminergic hyper-function are a widely accepted phenomenon of psychotic disorders [77], [78]. These neurotransmitter systems are closely linked since glutamate activates the inhibitory systems of dopamine and this regulatory response is necessary to maintain equilibrium of these neurotransmitters [79]. Antipsychotics are primarily dopamine antagonists which reduce dopamine levels but also increase glutamate levels [80]. Excessive activity of NMDA receptors has been implicated in mood disorders [81], [82] and treatment with mood stabilisers (i.e. lithium and lamotrigine) or antidepressants has been shown to reduce the glutamate levels associated with such activity [83]–[85]. A better understanding of the MMN profiles revealed in this study may help to determine whether different types of glutamatergic agents (if any) may be efficacious in treating certain individuals.

Although this is the first study using a data driven approach to determine MMN/P3a complex profiles in young people with psychiatric disorders, it is limited by its cross-sectional nature. For future studies, data at multiple time points across illness trajectory will help to better determine the temporal nature of illness. Additionally, by better defining stage and severity of illness the predictive capacity of these cluster profiles can be assessed. The cohort in this study was a sample of convenience and overall there are unequal ratios of primary psychotic, bipolar and depressive disorders; future studies should ideally have larger sample sizes with equal ratios of diagnostic category. Lastly, this analysis is exploratory and further research is warranted to replicate these findings.

This study highlights the utility of using a data driven method as an alternative to categorising individuals with psychiatric illnesses. In this respect, this study supports the notion that MMN and P3a are well suited to probe the underlying neurobiology of psychiatric disorders and provide important insights into the variations of neurochemical functions that appear to exist despite diagnostic category.
